# Enhancing saltiness perception by chemosensory interaction: an fMRI study

**DOI:** 10.1038/s41598-023-38137-2

**Published:** 2023-07-10

**Authors:** Afshin Faridi Esfanjani, Mohebbat Mohebbi

**Affiliations:** Department of Food Science and Technology, Ferdowsi University of Mashhad, Mashhad, Iran

**Keywords:** Feeding behaviour, Olfactory system

## Abstract

Neuroimaging studies that focus on taste, odor, and their interactions can specify their capability to elicit brain regions responsible for flavor perception and reward. Such information would be useful for formulating healthy food products, such as low salt food. In this study, a sensory experiment was conducted to investigate the capability of cheddar cheese odor, monosodium glutamate (MSG), and their interactions to enhance saltiness perception and preference of NaCl solutions. The activated brain areas in response to odor-taste-taste interactions were then investigated using an fMRI study. The results of the sensory tests showed that saltiness and preference of NaCl solutions were enhanced in the presence of MSG + cheddar cheese odor. According to the fMRI study, the stimulus with a higher salty rate activated the rolandic operculum, and the stimulus with a higher preference activated the rectus, medial orbitofrontal cortex, and substantia nigra. Moreover, the activation of multiple regions, such as the orbitofrontal cortex (OFC), anterior cingulate cortex (ACC), temporal pole, and amygdala was observed in response to (cheddar cheese odor + MSG + NaCl) minus (odorless air + NaCl).

## Introduction

Reducing salt content in food products without compromising taste is a significant challenge. To address this issue, it is essential to increase the perception of saltiness in the consumer's brain without increasing the NaCl concentration. Therefore, the efficacy of salty enhancer agents can be evaluated using neuroimaging techniques^1^. Sodium and chloride ions are released into the mouth when a food product is consumed, and these ions are transported to the taste buds, where taste receptors are present. These receptors produce chemical signals when activated, which are converted into electrical signals and sent to the brain via nerve fibers^2^. Salty perception can be influenced by other sensory experiences, such as taste and smell, through intermodal and crossmodal interactions^3^. Umami substances such as monosodium glutamate (MSG) and glutamate have been shown to have high potential in increasing saltiness perception and palatability of food products^4–6^. MSG has lower sodium content than NaCl, making it an attractive option for reducing sodium in food products^7,8^. Another strategy that has shown potential is the interaction of NaCl with congruent odors such as sardine, bacon, anchovy, tuna, ham, chicken, and soy sauce, to enhance the saltiness perception of low salt solutions^9^.

One critical step in developing low salt products is to measure the sensory discrimination and preference change of a developed product compared to the reference sample. Sensory discrimination tests based on a fixed reference are suitable for comparing sensory properties of different samples with a fixed reference product^10,11^. In recent years, the use of functional magnetic resonance imaging (fMRI) to evaluate activated brain areas during sensory experiences has become increasingly popular in the field of flavor chemistry. For example, García-González et al.^12^ used fMRI to study brain activity during the smelling of virgin olive oil of different qualities. They evaluated brain areas activated by unpleasant and pleasant odors through fMRI experiment^13^. The primary taste cortex is located in the anterior insula/frontal operculum, and the secondary cortical taste area is situated in the caudolateral orbitofrontal cortex (OFC)^14,15^. The anterior olfactory nucleus, pyriform cortex, rostral entorhinal cortex, amygdala, and periamygdaloid complex regions are the primary olfactory cortex. The secondary olfactory cortex is the orbitofrontal cortex, the cortical region receiving projections from the primary olfactory cortex^16,17^. There are overlapping in the region of the insula, operculum, OFC, and anterior cingulate cortex activated by taste or odor. Thus, the interaction of congruent odor and taste can enhance the activated area related to the intensity and preference of a taste^18^. Several studies had shown higher neural activation in multiple regions of the brain when congruent odor interacted with taste. For example, higher neural activation in different regions of the brain, including the anterior dorsal insula, anterior ventral insula/caudal OFC, anterior cingulate cortex (ACC), and posterior parietal, was observed when a complex of sweet taste and the retronasal vanilla odor was represented to subjects compared to an incongruent mixture of salty taste and retronasal vanilla odor^19^. Salty congruent odor (bacon)-0.16 M of NaCl showed higher activation in brain areas, such as bilateral anterior ventral insular, right inferior FO, right rolandic operculum, right ACC, superior frontal gyrus, angular gyrus, and claustrum in comparison with a mixture of incongruent odor (strawberry)-0.16 M of NaCl^20^. The neuroimaging studies investigated the perception of the interaction of salty congruent taste compounds, such as MSG with, NaCl^21^, and the interaction of salty congruent odors with NaCl^20^. Little is known about activated brain area by odor-taste-taste interactions. Therefore, the objective of this study is to use fMRI to investigate the neural activation in the brain when the salty congruent taste compound (MSG) and cheddar cheese odor interact with NaCl.

## Results

### Sensory analysis

**Figure 1:**
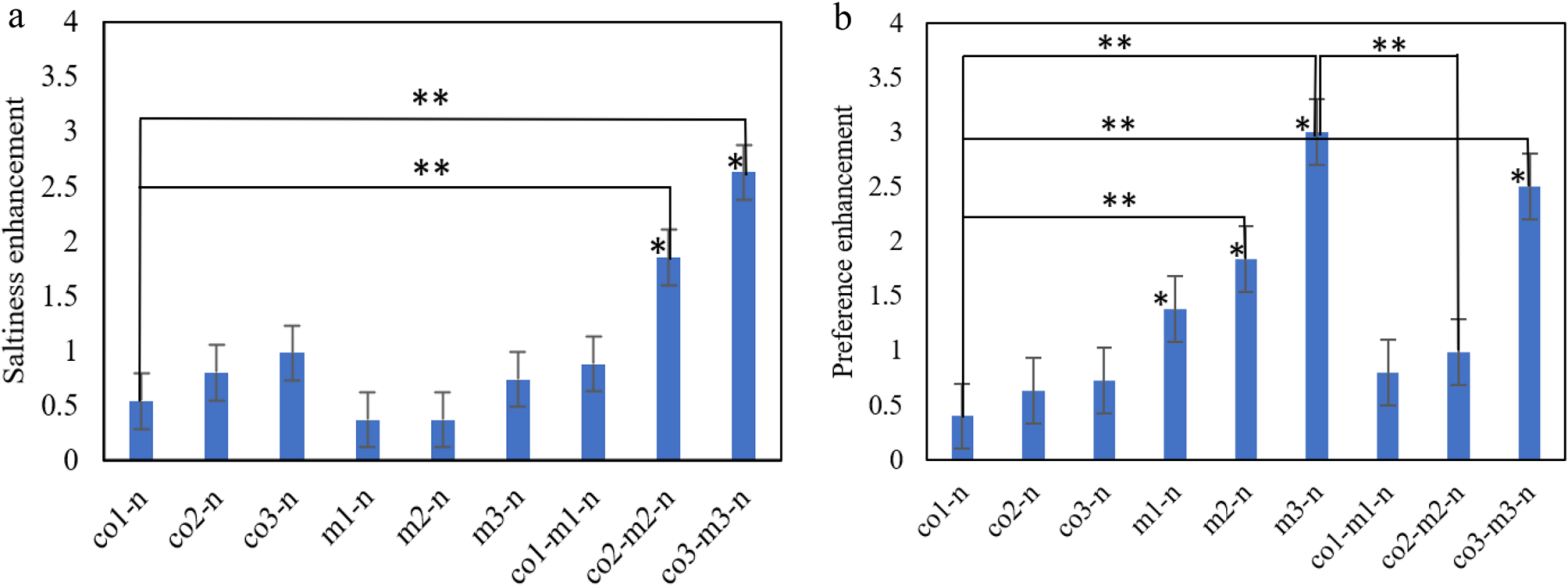
The impact of taste-taste, odor-taste, and odor-taste-taste interactions on saltiness perception (**a**) and preference (**b**). In the figure, "n" represents NaCl, "m" denotes MSG, and "co" refers to cheddar cheese odor. The levels of concentration for the ingredients are indicated by numbers 1, 2, and 3. Cheddar cheese odor concentrations were 0.0013, 0.0022, and 0.025 g/L, and MSG concentrations were 0.1, 0.17, and 0.5, respectively. In the figure, the symbol "*" shows statistical significance when compared to zero (p < 0.05), and "**" shows statistical significance when compared to other stimuli (p < 0.05).

As shown in Fig. 1, the saltiness perception and preference of test stimuli were compared with the NaCl + odorless stimulus. Our results showed that the saltiness perception of the stimulus containing 0.5 g/L NaCl + 0.5 g/L MSG + 0.025 g/L cheddar cheese odor or 0.0022 g/L produced higher saltiness than other stimuli (p < 0.05). In the case of the preference, NaCl solutions containing 0.5 g/L MSG or 0.5 g/L MSG and 0.025 g/L cheddar cheese odor produced higher preference than other stimuli (p < 0.05). Based on the measured saltiness perception and preference, the stimulus with the lowest saltiness intensity and preference was odorless air + NaCl solution (0.5 g/L). The stimulus with the highest saltiness intensity and preference among stimuli formulated with cheddar cheese odor + NaCl (odor-taste interactions) was cheddar cheese odor (0.025 g/L) + NaCl solution (0.5 g/L), while the stimulus with the highest saltiness intensity and preference among stimuli formulated with MSG + NaCl (taste-taste interactions) was odorless air + MSG (0.5 g/L) + NaCl (0.5 g/L). Finally, the stimulus with the highest saltiness intensity and preference among stimuli formulated with cheddar cheese odor + MSG + NaCl (odor-taste-taste interactions) was cheddar cheese odor (0.025 g/L) + MSG (0.5 g/L) + NaCl (0.5 g/L).

### fMRI analysis

The results of the behavioral tests indicated that there was no significant difference in saltiness perception between the combination of cheddar cheese odor and NaCl and the combination of odorless air, MSG, and NaCl (p > 0.05). However, the combination of odorless air, MSG, and NaCl was rated as more pleasant compared to the combination of cheddar cheese odor and NaCl (p < 0.05). Additionally, the combination of cheddar cheese odor, MSG, and NaCl was found to elicit higher perceptions of saltiness and preference compared to both the combination of cheddar cheese odor and NaCl and the combination of odorless air, MSG, and NaCl (p < 0.05). Table 1 shows activated brain areas toward (cheddar cheese odor + NaCl) and (odorless air + MSG + NaCl). (Cheddar cheese odor + NaCl) minus (odorless air + NaCl) activated different regions of the brain, including the middle frontal gyrus and middle temporal gyrus. Moreover, (odorless air + MSG + NaCl) minus (odorless air + NaCl) induced more brain areas, such as the superior frontal gyrus, inferior occipital gyrus, substantia nigra, etc. rectus, precentral gyrus, and substantia nigra (Table 1). Our results showed that when a NaCl interacting with MSG and cheddar cheese odor was presented to subjects, more areas of brain were activated compared to odorless air-NaCl, cheddar cheese odor-NaCl, and odorless air-MSG-NaCl as seen in Tables 2 and 3.

**Table 1:** Different activated brain regions toward (cheddar cheese odor + NaCl) and (odorless air + MSG + NaCl).

Brain region	Hemisphere	x	y	z	k	T value	p value
NC > NO							
Middle temporal gyrus	R	60	− 2	− 16	25	3.6	0.000
Middle frontal gyrus	R	42	50	0	51	3.83	0.000
Inferior frontal gyrus, opercular part	R	− 56	14	12	10	2.98	0.001
MNO > NO							
Superior frontal gyrus	L	− 22	− 8	74	37	3.25	0.000
Middle frontal gyrus	L	50	34	30	110	3.26	0.000
Rectus	L	− 10	36	− 20	23	3.59	0.000
	R	4	46	− 20	32	3.25	0.000
Precentral gyrus	L	− 38	− 22	56	20	3.07	0.001
Superior frontal gyrus, medial orbital	R	10	34	− 12	22	3.06	0.001
Inferior frontal gyrus, opercular part	R	44	10	24	34	2.93	0.002
Inferior occipital gyrus	R	32	− 90	− 8	54	347	0.001
Inferior parietal gyrus	L	− 44	− 56	48	46	3.2	0.000
Lobule IV, V of vermis		2	− 56	− 18	36	3.11	0.000
Substantia nigra, pars reticulata	L	− 6	− 18	− 20	21	3.56	0.000
MNO > NC							
Precentral gyrus	L	− 32	− 16	72	42	3.08	0.001
	L	− 36	− 12	64		2.81	0.002
	R	44	− 12	62	51	3.15	0.000
	R	36	− 16	66		2.81	0.002
Superior frontal gyrus, dorsolateral	L	− 20	4	54	41	2.86	0.002
Middle temporal gyrus	R	40	− 68	20	46	3.51	0.000
Calcarine fissure	L	− 2	− 82	12	44	3.31	0.000
Postcentral gyrus	L	− 54	− 14	50	23	3.06	0.001
Inferior frontal gyrus, opercular part	R	44	10	24	29	2.93	0.002
Posterior cingulate gyrus	L	0	− 48	22	26	2.96	0.002
Posterior cingulate gyrus	L	0	− 40	24		2.86	0.002
Substantia nigra, pars reticulata	R	− 12	− 16	− 14	13	2.89	0.002

Whole-brain analyses thresholded at uncorrected p < 0.005 and a minimum cluster size of 10 voxels; k, cluster size in voxels; xyz, MNI space peak.

**Table 2:** Different activated brain regions toward (odorless air + MSG + NaCl) minus (cheddar cheese odor + NaCl).

Brain region	Hemisphere	x	y	z	k	T value	p value
Superior frontal gyrus, dorsolateral	R	18	− 54	34	135	3.74	0.000
	R	42	− 48	− 28	35	3.54	0.000
	R	16	64	24	20	2.93	0.001
	R	− 6	− 86	− 30	37	3.22	0.000
Middle frontal gyrus	L	− 34	14	56	32	3.04	0.001
	R	50	6	− 20	156	3.50	0.000
Superior frontal gyrus, medial orbital	R	30	− 84	− − 32	64	4.32	0.000
Precentral gyrus	R	48	48	− 10	25	4.10	0.000
Inferior frontal gyrus, pars orbitalis	R	54	28	4	49	4.08	0.000
Inferior frontal gyrus, triangular part	R	32	− 76	46	317	4.07	0.000
Paracentral lobule	L	− 14	− 24	80	71	5	0.000
	L	46	− 72	− 34	54	5	0.000
Precentral gyrus	R	48	48	− 10	25	4.10	0.000
Angular gyrus	R	4	30	− 18	57	3.92	0.000
Rectus	R	− 44	− 54	58	74	3.89	0.000
	R	22	− 22	78	108	3.75	0.000
Temporal pole: superior temporal gyrus	L	60	4	8	39	3.09	0.000
	R	62	− 46	30	50	3.48	0.000
Temporal pole: middle temporal gyrus	R	2	58	− 4	119	4.34	0.000
SupraMarginal gyrus	R	− 38	− 36	66	92	3.48	0.000
Postcentral gyrus	L	4	− 2	4	42	3.41	0.000
Postcentral gyrus	L	− 48	− 20	56	212	3.31	0.000
Precentral gyrus	L	36	− 18	− 14	30	3.26	0.000
Hippocampus	L	60	− 8	42	663	4.14	0.000
	R	44	− 40	56	147	3.25	0.000
Inferior parietal gyrus	R	28	40	22	198	3.24	0.000
Posterior orbital gyrus	L	− 56	− 10	40	206	3.32	0.000
Middle cingulate & paracingulate gyri	L	− 30	− 82	− 32	86	3.66	0.000
Middle cingulate & paracingulate gyri	R	14	− 40	40	28	2.92	0.001
Anterior cingulate cortex, supracallosal	L	− 2	32	18	35	2.98	0.001
Anterior cingulate cortex, pregenual	R	4	44	0	21	2.82	0.002
Anterior cingulate cortex, supracallosal	R	24	− 64	− 24	56	3.23	0.000

Whole-brain analyses thresholded at uncorrected p < 0.005 and a minimum cluster size of 10 voxels; k, cluster size in voxels; xyz, MNI space peak.

**Table 3:** Different activated brain regions toward (cheddar cheese odor + MSG + NaCl) minus (odorless air + NaCl).

Brain region	Hemisphere	x	y	z	k	T value	P value
Superior frontal gyrus	L	− 16	2	58	49	3.3	0.000
	L	− 24	50	0	22	3.11	0.000
	R	16	32	52	156	4.14	0.000
	R	20	56	24	37	3.01	0.001
Middle frontal gyrus	L	− 32	4	62	68	3.34	0.000
	L	− 32	34	42	23	3.17	0.000
	R	44	48	4	30	3.3	0.000
	R	30	28	36	27	3.2	0.000
	R	− 32	34	42	23	3.17	0.000
	R	44	12	52	42	2.9	0.002
Precentral gyrus	L	− 30	− 18	72	56	3.8	0.000
	L	− 36	− 10	62	106	3.52	0.000
	R	62	6	24	32	4	0.000
	R	48	10	26	42	3	0.001
Inferior frontal gyrus, triangular part	L	− 52	32	0	30	3.73	0.000
	R	58	22	6	72	3.18	0.000
OFCpost		− 34	34	− 20	77	3.87	0.000
OFCant		20	52	− 12	31	3.57	0.000
Inferior frontal gyrus, pars orbitalis	R	48	48	− 10	84	3.68	0.000
Rolandic operculum	L	− 48	2	14	49	3.42	0.000
Supplementary motor area	L	− 4	− 16	62	64	3.37	0.000
	R	6	0	54	32	3.26	0.000
Rectus	L	2	32	− 18	34	3.25	0.000
Superior frontal gyrus, medial gyrus	R	4	54	10	63	3.72	0.000
Superior frontal gyrus, medial orbital	R	4	58	− 4	30	3.24	0.000
Posterior orbital gyrus	L	− 34	34	− 20	77	3.87	0.000
Anterior orbital gyrus	R	20	52	− 12	31	3.57	0.000
Amygdala	L	− 16	0	− 14	26	4.68	0.000
Hippocampus	L	− 20	− 12	− 12	31	3.79	0.000
Middle cingulate & paracingulate gyri	L	0	− 44	34	84	3.26	0.000
Paracentral lobule	L	− 14	− 24	80	39	4.97	0.000
	L	− 4	− 16	64	25	3.1	0.000
Inferior occipital gyrus	L	− 20	− 98	− 10	30	3.42	0.000
Inferior parietal gyrus	R	58	− 40	50	24	3.3	0.000
Precuneus	L	− 6	− 62	40	31	3.07	0.001
	R	24	− 44	6	25	4.10	0.000
	R	16	− 52	34	32	3.09	0.000
Temporal pole: middle temporal gyrus	R	38	16	− 34	140	4.74	0.000
Temporal pole: superior temporal gyrus	R	50	6	− 20	108	4.44	0.000
	R	34	6	− 22	37	3.389	0.000
Middle temporal gyrus	L	− 52	− 22	− 18	20	3.15	0.000
	L	− 52	− 70	20	75	3.62	0.000
Anterior cingulate cortex, pregenual	R	4	50	8	90	3.75	0.001
Anterior cingulate cortex, supracallosal	L	0	26	18	29	3.01	0.000
	R	16	36	22	26	3.01	0.001
Substantia nigra, pars reticulata	L	− 12	− 20	− 14	24	3.35	0.000

Whole-brain analyses thresholded at uncorrected p < 0.005 and a minimum cluster size of 10 voxels; k, cluster size in voxels; xyz, MNI space peak.

## Discussion

The association of orthonasal odor and taste is important in the eating process. Humans often try to smell food via an orthonasal route before eating a food product. This study examines the odor-taste interactions during the initial step of eating. Since odors were delivered via an orthonasal route, the possibility of odor-taste interactions in the mouth was low. Therefore, enhancing saltiness perception by a congruent odor does not occur at a peripheral level but at a central level of processing^20^. Moreover, MSG is one of the popular compounds used to reduce sodium content in several studies^7^; increase saltiness perception and palatability of NaCl solutions^5^, and use umami carriers, such as MSG, to enhance levels of saltiness perception of salt solutions^22^. Thus, in the present study, a mixture of MSG and cheddar cheese odor was used to enhance the saltiness perception of NaCl solutions. Our fMRI study showed that (cheddar cheese odor + MSG + NaCl) minus (odorless air + MSG + NaCl) strongly activated rolandic operculum (Table 3). Frontal operculum makes up the taste cortex (TC), which encodes the features of pure taste stimuli, such as quality and intensity^23^. Indeed, stronger activation of operculum toward (cheddar cheese odor + NaCl) showed a higher perception of saltiness. The perception of a tastant can be increased in the presence of a congruent odor since independently presentation of a tastant or an odorant produces overlapping activation in some regions of the brain, such as operculum^18^. Thus, as shown in results of the sensory experiments, cheddar cheese odor + MSG + NaCl produced higher saltiness perception. In the case of preference of NaCl solutions, Seo et al.^20^ showed that the NaCl solution alone did not have a pleasant taste. Also, their results showed more disliking to the congruent odor-induced saltiness enhancement in the NaCl solution. In our study, the preference for test stimuli was compared to the preference for 0.5 g/L NaCl + odorless air. In the present study, a low concentration of NaCl was used to prepare salty solutions, which were not too saline. Our results in Fig. 1 showed that the NaCl solution containing cheddar cheese odor produced a higher preference than zero (the preference of NaCl + odorless air), but it was not significant (p > 0.05). However, the NaCl solution containing MSG produced a higher preference than zero (p < 0.05). This can be related to the pleasant activity of MSG. MSG has lower sodium content than NaCl, making it an attractive option for reducing sodium in food products^7,8^. According to Fig. 1b, adding 0.5 g/L MSG to NaCl solutions and NaCl + 0.025 g/L cheddar cheese odor produced more preference than other stimuli (p < 0.05). This showed that 0.5 g/L MSG had higher activity to enhance the preference for NaCl solution than 0.17 g/L and 0.1 g/L MSG. In our neuroimaging study, we observed distinct patterns of brain activation. Specifically, the contrast between odorless air combined with MSG and NaCl, compared to odorless air combined with NaCl alone, resulted in the activation of brain regions such as the rectus and substantia nigra. Additionally, when comparing odorless air combined with MSG and NaCl to cheddar cheese odor combined with NaCl, activation in the posterior cingulate gyrus and substantia nigra was observed. These regions are associated with feelings of pleasantness, indicating that the addition of 0.5 g/L MSG to NaCl solutions can generate increased levels of pleasantness. Moreover, in the present study, substantia nigra compacta was strongly activated by (cheddar cheese odor + NaCl) minus (odorless air + NaCl). The substantia nigra compacta contains dopaminergic neurons and is activated toward pleasant tastes^24^. Precentral gyrus and precuneus were strongly activated by (cheddar cheese odor + MSG + NaCl) in the present study. The precuneus is functionally connected to reward regions (e.g., striatum and midbrain)^25^. The precentral gyrus is a motor planning region and elevated activation in this region in response to food cues has been interpreted as potentially reflecting motor planning to consume food^26^. Also, (cheddar cheese odor + MSG + NaCl) minus (odorless air + NaCl) activated temporal pole. The medial temporal-lobe has a functional role in olfaction, which is associated with the intensity and not with the affective valence of odors^27^. Rectus, ACC, and substantia nigra were strongly activated by (cheddar cheese odor + MSG + NaCl) minus (odorless air + NaCl). Moreover, amygdala and hippocampus were strongly activated in response to (cheddar cheese odor + MSG + NaCl) minus (odorless air + NaCl); These parts of the brain have many neural connections with OFC^28^. Studies showed that amygdala could be activated by either pleasant taste (e.g., glucose) or unpleasant taste (e.g., saline)^29^. Considering the higher preference observed for the combination of cheddar cheese odor (0.022 g/L), MSG (0.5 g/L), and NaCl (0.5 g/L) in the sensory experiment, the activation of the rectus, amygdala, ACC, and substantia nigra indicated a stronger preference for the combination of cheddar cheese odor, MSG, and NaCl. In a similar study by McCabe and Rolls^30^, more pleasantness was obtained by combining (vegetable odor + MSG) compared to MSG. Pleasantness ratings of MSG + vegetable odor were reflected in the medial orbitofrontal and pregenual cingulate cortex activations. As seen in Table 2, anterior and posterior orbitofrontal cortex were activated in response to (cheddar cheese odor + MSG + NaCl). In a study by Rolls^31^, more activation of an anterior part of the orbitofrontal cortex was observed when MSG combined with inosine 5′-monophosphate (IMP). This showed that MSG solutions produced a higher umami taste perception in the presence of IMP.

In our fMRI study, we aimed to explore the potential of combining cheddar cheese odor and MSG in enhancing the perception of low salt solutions (0.5 g/L NaCl). We compared the brain activations between (cheddar cheese odor + MSG + NaCl) minus (odorless air + NaCl) and odorless air + MSG + NaCl conditions. Multiple brain regions exhibited activation in response to the combination of cheddar cheese odor, MSG, and NaCl, including the anterior and posterior orbitofrontal cortex, rolandic operculum, ACC, frontal gyrus, middle temporal gyrus, rectus, substantia nigra, amygdala, hippocampus, contrast to the odorless air + MSG + NaCl condition. This finding suggests that when combined with MSG and cheddar cheese odor, NaCl solutions elicited a higher perception of saltiness and preference. Based on these results, it can be concluded that the combination of MSG, a congruent odor (cheese odor), and NaCl could be an effective approach for developing healthy low-salt food products. This combination has the potential to enhance the perception of saltiness while reducing the actual salt content.

### Limitations

The number of subjects was low in the present study because sensory experiments were conducted in the Covid-19 era, which was a limitation of this study. Additionally, the current threshold used at the second level analysis for fMRI is inadequate, and it is crucial to employ a multiple comparison correction approach, such as the family-wise error rate, to ensure accurate results. Since the association of orthonasal odor and taste is important in the eating process, humans look at the food and often try smelling that via an orthonasal route before eating a food product. Therefore, the present study examined the odor–taste-taste interactions with a focus on the initial step of eating. However, future studies could focus on odor delivery from retronasal route to interact with taste compounds.

## Material and methods

### Ethics approval and consent to participate

All volunteers gave informed consent before participating. This study was conducted following the declaration of Helsinki. This project was approved by reviewed and approved by the Research Ethics Committees (REC) of Ferdowsi University of Mashhad (FUM). IR.UM.REC.1400.129.

### Stimuli delivery systems

Controlled olfactometer and gustometer were used to deliver odor and taste stimuli during sensory experiments and fMRI experiment. Their construction was described in our previous study^32^.

### Sensory experiment

A total of twelve subjects (with ages ranging from 19 to 40 years; body mass index BMI 21–32) were recruited for the study, including five males and seven females. In the first experiment, the sensory analysis was performed in three seasons. First, all study steps with details were explained to subjects, and they were screened to confirm that they had no clinical history of major diseases and that they had normal smell and taste functions. Then, the threshold points of each stimulus, including NaCl, MSG, and cheddar cheese odor, were calculated. In the final step, the saltiness intensity and preference of each stimulus, including MSG + NaCl, cheddar cheese odor + NaCl, and cheddar cheese odor + MSG + NaCl, were analyzed.

### Stimuli

Stimuli including MSG-NaCl, cheddar cheese odor-NaCl, and cheddar cheese odor-MSG-NaCl were prepared according to Table 1. 0.5 g/L of NaCl concentrations (above the threshold) interacted with three concentrations (below the threshold, equal to the threshold, above the threshold) of each MSG and cheddar cheese odor. According to our previous study Esfanjnai and Mohebbi^32^, threshold points of NaCl, MSG, and cheddar cheese odor were 0.31 g/L, 0.17 g/L, and 0.0022 g/L, respectively. To determine the threshold, subjects were exposed to varying concentrations of stimuli. The threshold of NaCl and MSG was measured using a two-alternative forced-choice method. Initially, solutions of NaCl and MSG at concentrations of 0.0625, 0.25, 0.5, 1, and 2 g/L were prepared. Subsequently, 10 mL of each solution was provided to the subjects in plastic glasses. Alongside each solution, a glass of tasteless water was also given to the subjects. The subjects were then asked to taste the stimuli and select the glass with the discernible taste. During the interstimulus interval, the subjects rinsed their mouths with water. For investigating the threshold of odors, 0, 0.000025, 0.00025, 0.0025, 0.025, 0.25 g/L solutions of cheddar cheese odor (OlbrichtArom, Germany) were selected. Participants were seated on a chair. Using the method of ascending limits, odors stimuli (from lowest concentration to highest concentration) of 3000 ms duration were presented to both nostrils at the rate of 6 L/min (35 ± 2 °C, humidity > 60%) using the computer-controlled olfactometer. Subjects were asked to answer “yes” or “no” whether an odor had been perceived or not. When participants perceived the odor at a certain concentration, they received the same concentration a second time. The threshold level was defined as the concentration where a given stimulus had been perceived twice in a row. The proportion of responses was depicted against concentrations of stimulus. Then, these data were fitted by the cumulative distribution function of the Weibull programmed on MATLAB version 7.5.0.342 (R2007b). The following equations define the cumulative distribution function of the Weibull parameters. Linear regression was used to fit the Weibull distribution.

$$F\left(x;\alpha :\beta \right)=1-\mathrm{exp}\left[-{\left(\frac{x}{\alpha }\right)}^{\beta }\right], 0 \le \mathrm{ x }<\mathrm{ \infty }.$$

α is a threshold (the percentage of stimuli that 81% of participants perceive), and β is the slope. Therefore, in the present study, the threshold of gustatory and olfactory was defined as the concentration at which the probability of detection was 81%.

### Analyzing saltiness perception and preference

**Figure 2:**
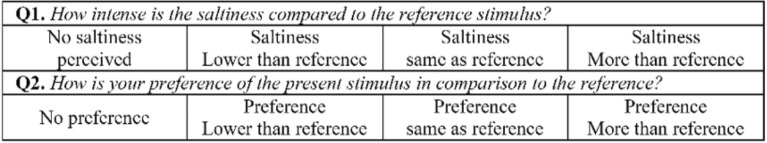
Questions for the evaluation of saltiness and preference of the stimuli.

Stimuli were presented to subjects according to Table 4. The delivery tube of the olfactometer was inserted into both nostrils of the subjects, and the delivery tube of the gustometer was incorporated into the subjects’ tongues. The orthonasal route was considered to deliver odor agents to subjects in this study since humans try to smell food before consuming food. Cheddar cheese odor-or odorless air was presented to subjects at the rate of 6 L/min (35 ± 2 °C, humidity > 60%) for 2 s. Subsequently, 3 mL of a taste solutions, including (NaCl)-or (NaCl + MSG), were delivered to subjects’ tongues (30 mL/min, 6 S). Then, subjects were asked to evaluate each test stimulus in the Table 4 by ranking the perceived saltiness and preference, as shown in Fig. 2. All stimuli were presented to subjects randomly. The interstimulus interval was 2 min, and the subject’s tongue was rinsed with water. NaCl + odorless was introduced as a reference stimulus for subjects. Then, they were asked to compare saltiness perception and preference of stimuli according to Fig. 2. Saltiness/preference enhancement corresponded, for each subject, to the difference between (i) the saltiness of the test stimulus and (ii) the reference.

**Table 4:** The formulation of stimuli based on the interaction of cheddar cheese odor-MSG-NaCl.

Number	Code	NaCl (g/L)	MSG (g/L)	Cheddar cheese odor (g/L)	Odorless air
1	a-n	0.5	–	–	✓
2	m1-n	0.5	0.1	–	–
3	m2-n	0.5	0.17	–	–
4	m3-n	0.5	0.5	–	–
5	co1-n	0.5	–	0.0013	–
6	co2-n	0.5	–	0.0022	–
7	co3-n	0.5	–	0.025	–
8	co1-m1-n	0.5	0.1	0.0013	–
9	co2-m2-n	0.5	0.17	0.0022	–
10	co3-m3-n	0.5	0.5	0.025	–

n is NaCl, a is odorless air, m is monosodium glutamate, co is cheddar cheese odor and 1, 2, 3 specify the level of concentration of the ingredients.

### fMRI experiment

#### Participants

Twelve adult participants (20–45 years) were recruited for the fMRI experiment, including eight males and three females. Before maim experiments, all study steps with details were explained to participants. All participants had no clinical history of major diseases and have normal gustatory and olfactory functions according to self-reports.

#### Stimuli

In this stage, four stimuli were chosen based on their saltiness and preference intensity as determined in the sensory experiment. Sample number 1 consisted of 0.5 g/L NaCl combined with odorless air, representing the stimulus with the lowest saltiness intensity and preference. Sample number 7 comprised 0.5 g/L NaCl combined with 0.025 g/L cheddar cheese odor, which exhibited higher saltiness intensity and preference compared to other stimuli involving cheddar cheese odor + NaCl (representing odor-taste interactions). Sample number 4 included 0.5 g/L NaCl combined with 0.5 g/L MSG, demonstrating higher saltiness intensity and preference compared to other stimuli involving MSG + NaCl (representing taste-taste interactions). Lastly, sample number 10 encompassed 0.5 g/L NaCl combined with 0.5 g/L MSG and 0.025 g/L cheddar cheese odor, exhibiting higher saltiness intensity and preference compared to other stimuli involving cheddar cheese odor + MSG + NaCl (representing odor-taste-taste interactions).

### fMRI experimental design

**Figure 3:**
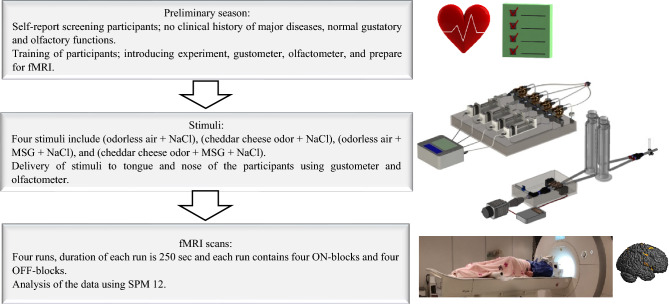
Schematic drawing of experimental design.

**Figure 4:**
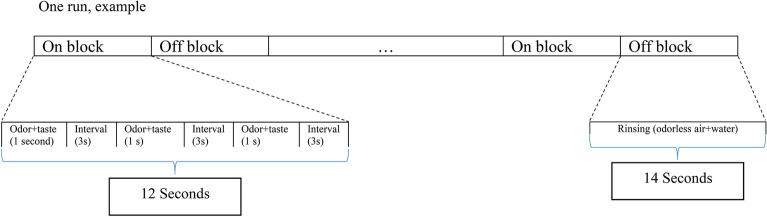
Schematic drawing of fMRI seasons including four runs in a block-designed.

Taste compounds were NaCl and MSG, and odor compounds were cheddar cheese odor and odorless air. As mentioned in the last section, four stimuli, including (odorless air + NaCl), (cheddar cheese odor + NaCl), (odorless air + MSG + NaCl), and (cheddar cheese odor + MSG + NaCl), were presented to participants. Taste solutions were delivered to the tongue by a gustometer, and odors were delivered to two nostrils of subjects by an olfactometer. The parts of the gustometer and olfactometer containing metal pieces were located outside of the fMRI room, and tastants and odors were delivered to subjects by 7 m of polyurethane tubes. A four-run block design was used for the fMRI experiment. In each run, four ON-blocks and four OFF-blocks were built. During ON-block, one of the four stimuli was presented to subjects (stimulus duration: 1 s; the interval between stimuli: 3 s) three times. At each time, 0.4 mL taste and odor (1 s at 2 mL/min) were delivered to subjects. During every off-block, tasteless water (4 mL) and odorless air (4 s at the rate of 2 mL/min) were presented to participants to minimize the residual effect of previous stimuli. In each session stimuli conditions were randomized. The instructions were visually presented to the subjects during the test. In the ON-blocks, they were instructed to hold the liquid in their mouths, while in the OFF-blocks, they were instructed to swallow it. A sketch of a single fMRI session is shown in Fig. 3 and the entire experimental design is depicted in Fig. 4. After each run, participants randomly received one of stimuli, and they were verbally asked to rate saltiness intensity (0 = extremely weak; 10 = extremely strong) and preference of each stimulus (− 5 = extremely unpleasant; + 5 = extremely pleasant).

### fMRI data acquisition and analyses

The experiment was done in the National Brain Mapping Laboratory, Iran. A 3 Tesla Siemens Prisma MRI scanner was used to acquire fMRI data. Functional images were acquired with repetition time = 2500 ms, echo time = 40 ms, flip angle = 90°, matrix = 64 × 64, and voxel size = 3 × 3 × 3.75 mm^3^. After the functional runs, high-resolution (1 × 1 × 1 mm^3^). T1-weighted anatomical images were acquired by a ‘‘magnet prepared rapid gradient echo’’ sequence with the following parameters: TR = 1.89 s, slice thickness = 1 mm, slice gap = 0. SPM12 (Wellcome Trust Centre for Neuroimaging, London, UK) implemented in MATLAB 2017b (MathWorks, Natick, MA, USA) was used to analyze the fMRI data. Spatial preprocessing included realignment, co-registration of functional with anatomical images, segmentation, normalization, and smoothing (8 × 8 × 8 mm^3^ FWHM Gaussian kernel)^33^. OFF-blocks were not included in data analyses. The 2 × 2 factorial design we used: odor (present or absent), and MSG (present or absent). To identify activated areas in general, the Automated Anatomical Labeling (AAL) tool in the SPM12 framework was used.

In the conducted sensory experiment, a 2-sample t-test was employed to investigate and establish statistical differences in both saltiness perception and preference between two distinct stimuli. The objective was to assess whether there were significant variations in how participants perceived the saltiness and expressed their preferences for the two stimuli being compared. For analyzing behavior data, a fully randomized design with a factorial arrangement involving two factors: odor (present or absent) and MSG (present or absent) was used. To analyze the data, Minitab 19 software was employed, and the significant differences between the means were determined using the Tukey test at a confidence level of 95% (p < 0.05). Minitab 19 software was used to analyze the data.

## Data Availability

The datasets used and/or analyzed during the current study available from the corresponding author on reasonable request.
